# Recovery of Homonymous Hemianopsia Following the Clipping of a Large Thrombosed Anterior Cerebral Artery Aneurysm Compressing the Optic Tract: A Case Report

**DOI:** 10.7759/cureus.70122

**Published:** 2024-09-24

**Authors:** Takashi Iimori, Yasuaki Inoue

**Affiliations:** 1 Department of Neurosurgery, Nadogaya Hospital, Chiba, JPN

**Keywords:** anterior cerebral artery aneurysm, cerebral aneurysm surgery, compressive optic neuropathy, microsurgical aneurysm clipping, visual field defect

## Abstract

Unruptured cerebral aneurysms can cause visual dysfunction by compressing the optic pathways. However, there are no reports of an aneurysm at the A1 segment of the anterior cerebral artery compressing the optic tract. A 69-year-old male presented with progressive right homonymous hemianopsia, and radiological examinations revealed a large, partially thrombosed left A1 aneurysm compressing the left optic tract. The patient underwent microsurgical clipping of the aneurysm, which significantly resolved the visual field defect. In cases of progressive visual dysfunction, aneurysms should be considered in the differential diagnosis. Early surgical decompression is important for optimal visual outcomes, and microsurgical clipping may be the most effective treatment.

## Introduction

Unruptured cerebral aneurysms can cause visual dysfunction by compressing the optic pathways. Commonly reported sites of aneurysms associated with visual symptoms include the cavernous portion of the internal carotid artery (ICA), the ophthalmic portion of the ICA, and the anterior communicating artery (ACoA). These aneurysms typically compress the optic nerve or the chiasm while cases involving compression of the optic pathways posterior to the chiasm are very rare [1]. Aneurysms originating from the A1 segment of the anterior cerebral artery (ACA) account for less than 1% of all intracranial aneurysms and tend to rupture at a smaller size than those from other arteries, often before they produce symptoms [2]. We report the first case of an unruptured A1 aneurysm compressing the optic tract, presenting with homonymous hemianopsia.

## Case presentation

**Figure 1:**
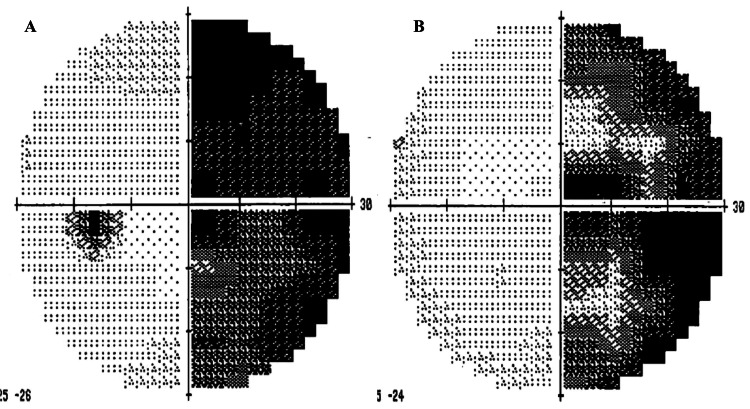
Perimetry charts of the Humphery two months before the surgery A, B: Perimetry charts of the Humphery demonstrating right homonymous hemianopsia (A (left eye), B (right eye): two months before the surgery)

A 69-year-old man came to our outpatient office with worsening vision disturbances for nine months. He initially visited another clinic to see an ophthalmologist, where no ophthalmologic abnormalities were revealed except for a right homonymous hemianopsia (Figures 1A, 1B).

He had a history of hypertension treated with a calcium channel blocker. Laboratory data on admission were notable for mild hyperlipidemia. Neurological examination was remarkable for a right homonymous hemianopsia.

**Figure 2:**
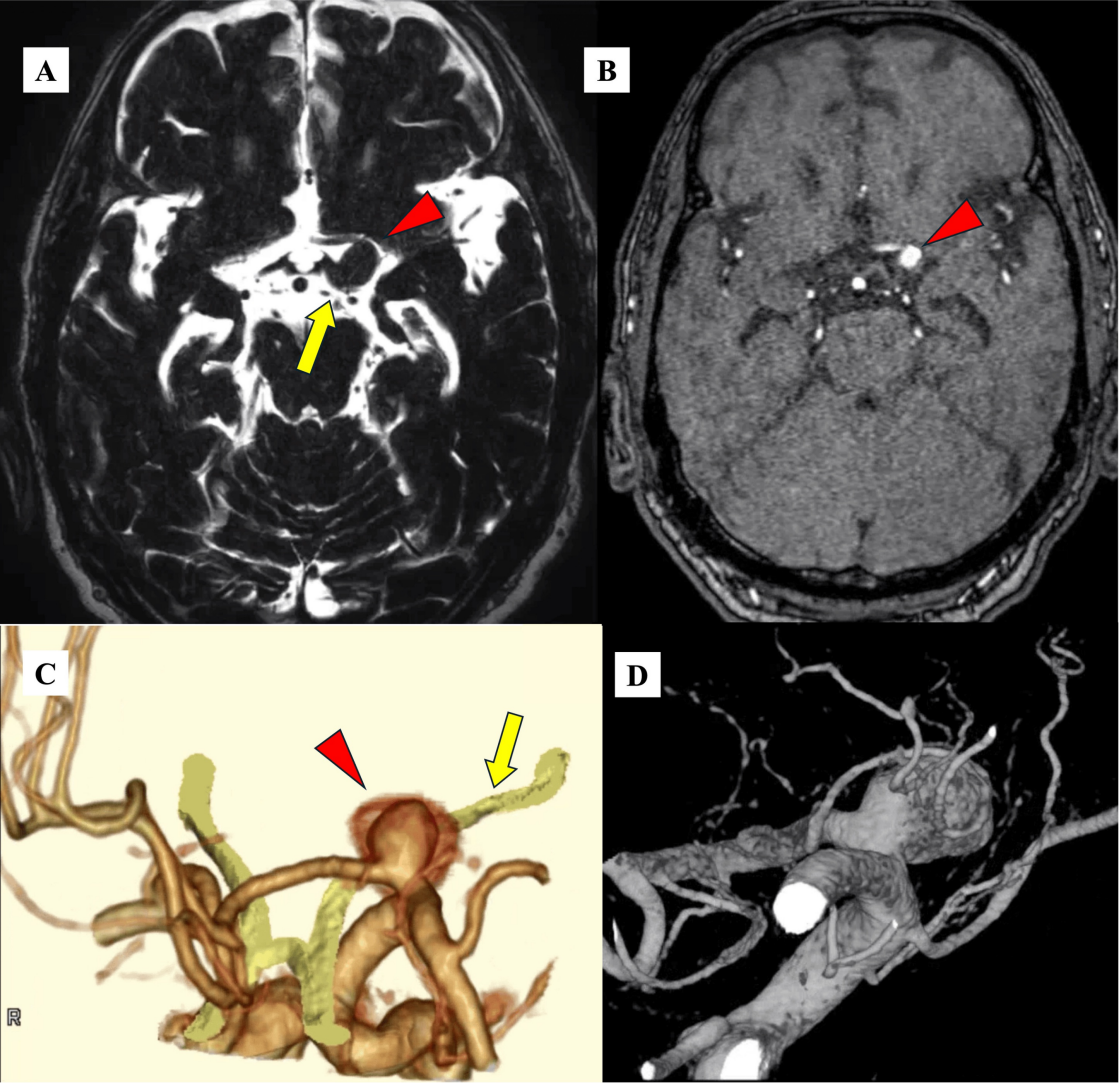
Preoperative images of the A1 aneurysm A, B: magnetic resonance (MR) cisternography and MR angiography revealing a thrombosed A1 aneurysm (red arrowhead) compressing the left optic tract (yellow arrow) from the anterior lateral side. C: 3D reconstruction of computed tomography angiography showing the aneurysm (red arrowhead) compressing the left optic tract (yellow arrow) from the anterior lateral side. D: 3D reconstruction of the left internal carotid angiography showing the A1 aneurysm projecting posteriorly and no perforators arising from the neck of the aneurysm.

Brain magnetic resonance imaging (MRI) and brain computed tomography (CT) angiography revealed a 15 mm partially thrombosed unruptured saccular aneurysm arising from the A1 segment of the left ACA (Figures 2A, 2B). The aneurysm was projecting posteriorly, compressing the left optic tract from the anterior lateral side (Figure 2C). We offered a clipping surgery for the aneurysm rather than an endovascular treatment, expecting the effect of clipping to reduce the mass effect. We preoperatively performed a cerebral angiogram to confirm that no perforators were arising from the neck (Figure 2D).

**Figure 3:**
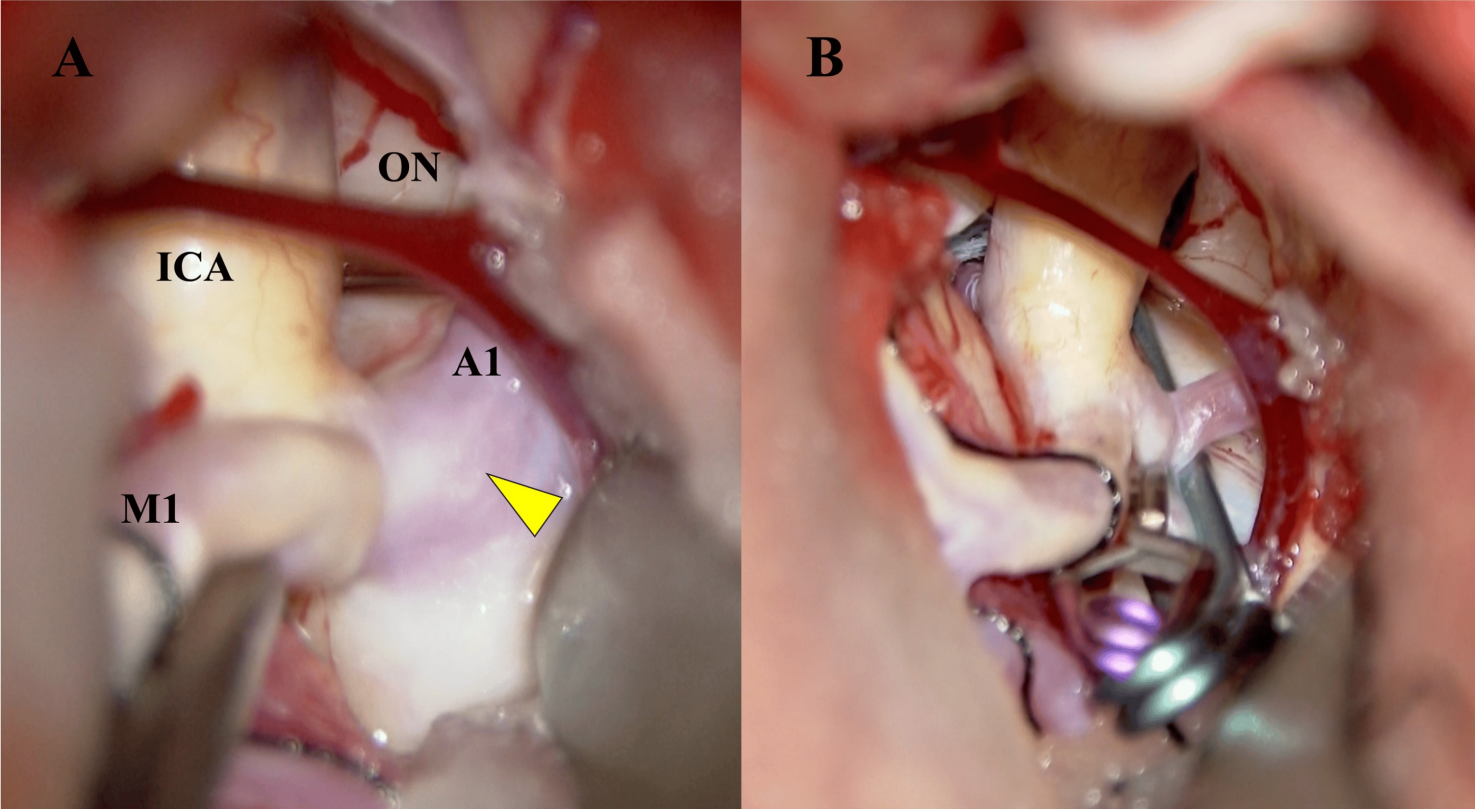
Intraoperative findings obtained before (A) and after (B) aneurysmal neck clipping A: The neck of the aneurysm was identified (yellow arrowhead), and perforators were detached from it. B: A Sugita clip (No. 12) and a Yasargil clip (FT692T) were applied to the neck to obliterate the aneurysm.

This surgery was performed 11 months after the onset of the symptoms. A left pterional craniotomy for the trans-sylvian approach was performed. Intraoperatively, the neck of the aneurysm was identified, and perforators were carefully detached from it. A Sugita clip (No. 12) and a Yasargil clip (FT692T) were applied to the neck to obliterate the aneurysm (Figures 3A, 3B).

**Figure 4:**
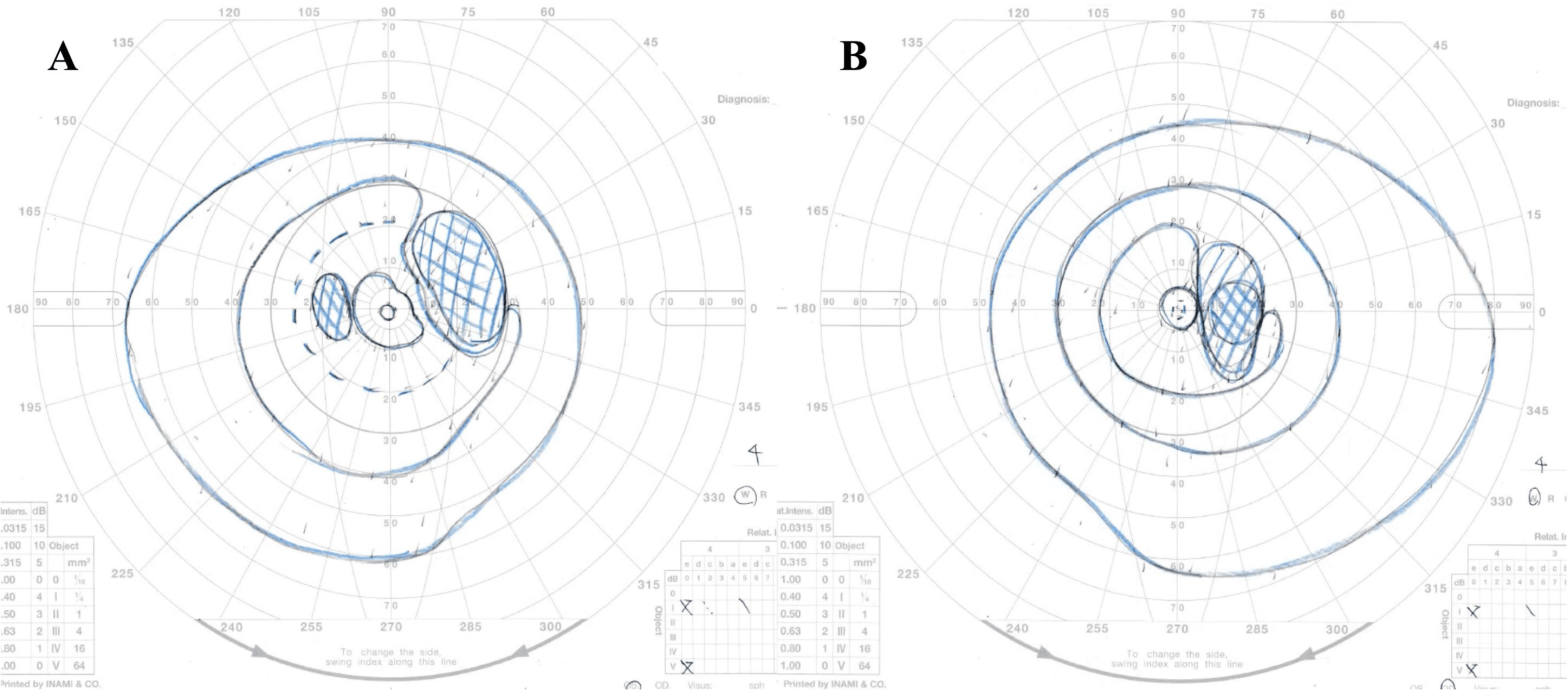
Perimetry charts of the Goldmann three days after the surgery A, B: Perimetry charts of the Goldmann demonstrating remarkable improvements of the visual field (A (left eye), B (right eye): three days after the surgery)

The patient had his visual field defect remarkably improved by day three after the surgery with no new neurological deficits (Figures 4A, 4B).

## Discussion

To the best of our knowledge, this report presents the first case of a thrombosed large A1 aneurysm compressing the optic tract. Similar cases have been reported in which aneurysms of ACoA or ICA compressed the optic chiasm, resulting in homonymous hemianopsia [3,4]. Aneurysms originating from the A1 segment of ACA are relatively rare but can be challenging to treat due to their location and proximity to perforators. Both microsurgical and endovascular treatments can achieve good outcomes with careful consideration of aneurysm characteristics and patient factors [2,5]. In particular, A1 aneurysms projecting posteriorly, as seen in our case, showed a favorable prognosis with either approach [5]. It is also important to compare postoperative visual outcomes among treatment methods, although the optimal approach for patients with visually symptomatic aneurysms remains uncertain.

The mechanisms of visual defects caused by aneurysms are commonly explained by two theories. First, direct compression of the optic pathway can cause visual impairments through the blockage of conduction [1,6]. Second, the blood supply of the optic pathway can also be compromised by the compression of the ophthalmic artery or small arteries [7].

De Oliveira et al. reported microsurgical clipping with a reduction in aneurysmal mass, which improved visual dysfunction caused by optic nerve compression in 93.3% of the cases [8]. Schuss et al. also reported that 75% of patients who received clipping surgery achieved improvement in visual symptoms, compared with 38% of patients who received endovascular treatment [9]. There have been reports of improvement in visual field defects after endovascular treatment [9]. However, coil embolization is reported to be sometimes associated with visual deterioration, which might be caused by progressive mass effects from thrombosis of aneurysms or perianeurysmal inflammation and edema [7]. Flow diversion (FD) has recently been introduced as an alternative treatment for large aneurysms. FD has been shown to result in aneurysm shrinkage [10] and effective recovery from visual symptoms caused by the mass effects of the aneurysm [11]. Some reports showed its superiority in the effects of vision disturbances to clipping and coiling for large paraclinoid aneurysms [12]. However, the complete occlusion rate for giant or thrombosed aneurysms is still lower than microsurgical clipping [10], and there is still limited evidence to guide treatment options. In this case, we offered a clipping surgery for the aneurysm rather than endovascular treatment, considering the superior occlusion rates associated with clipping for thrombosed aneurysms and its greater potential to alleviate the mass effect on the optic tract.

Surgical treatment for aneurysms within three to eight months after the onset of symptoms has been reported to be associated with good visual outcomes and the importance of early treatment is emphasized [1,8,13]. On the other hand, some studies showed no significant relationship between recovery from visual dysfunction and the duration of visual symptoms [9,14]. There are reports of favorable visual outcomes in patients treated for aneurysms causing visual defects, despite the symptoms persisting for more than 12 months [9]. In the present case, the timing of surgical intervention was impacted by the patient’s delayed consultation with the neurosurgeon and challenges in coordinating the surgical schedule. Nevertheless, the visual field recovered despite the aneurysm being treated 11 months after symptom onset. It remains uncertain whether earlier intervention or alternative treatment methods could have resulted in better visual outcomes. Factors for good visual outcomes have been still unclear and further evidence needs to be accumulated.

Limitations of our case report include inconsistencies in the visual field assessments conducted before and after treatment, as the pre-treatment evaluation was performed at another clinic rather than at our hospital. For clearer comparisons, identical visual field assessment methods should have been used.

## Conclusions

We described, to the best of our knowledge, the first reported case of an A1 aneurysm compressing the optic tract and presenting with homonymous hemianopsia. It is important to include an unruptured aneurysm in the differential diagnosis of patients who experience visual dysfunction. In patients with progressive visual field loss caused by an aneurysm, removing aneurysmal mass effects on the optic pathway is crucial for achieving optimal visual outcomes, and microsurgical clipping is an effective treatment.
